# Robot-Assisted Diaphragmatic Plication in an Adult Bochdalek Hernia With Intrathoracic Hepatic Displacement

**DOI:** 10.1016/j.atssr.2025.09.009

**Published:** 2025-10-09

**Authors:** Marco N. Andreas, Alessandro Panelli, Moritz Gross, Johann Pratschke, Jens C. Rückert, Aron Elsner

**Affiliations:** 1 Department of Surgery, Charité–Universitätsmedizin Berlin, Berlin, Germany; 2 Department of Anesthesiology and Intensive Care Medicine, Charité–Universitätsmedizin Berlin, Berlin, Germany; 3 Department of Radiology, Charité–Universitätsmedizin Berlin, Berlin, Germany

## Abstract

We report the case of a 72-year-old woman with a 9-month history of dyspnea and impaired gas exchange. The patient had significant cardiac comorbidities. Computed tomography imaging revealed right diaphragmatic elevation with intrathoracic displacement of the liver (in a 90° angle). A robot-assisted diaphragmatic plication was performed. Intraoperative contrast-enhanced ultrasound was performed to rule out hepatic torsion and confirmed preserved perfusion. This case underscores the importance of accurate diagnosis and multidisciplinary planning in adult Bochdalek hernia with hepatic herniation. Robot-assisted repair with perfusion monitoring was found to be safe and effective.

Bochdalek hernias are congenital diaphragmatic defects resulting from failure of the posterolateral pleuroperitoneal canal to close during embryogenesis.^1^ Whereas they are typically diagnosed in neonates because of respiratory distress caused by pulmonary hypoplasia, adult presentations are rare.^2^

Right-sided Bochdalek hernias are particularly uncommon, mainly owing to the protective position of the liver, and are more likely to be associated with visceral herniation. In such cases, surgical intervention is warranted to prevent complications such as organ torsion and compromised perfusion. Minimally invasive approaches offer improved visualization and precision, particularly in anatomically complex cases.^3^

A 72-year-old woman presented with progressive dyspnea during a 9-month period and evidence of impaired gas exchange. She had a history of mini–gastric bypass surgery. Computed tomography imaging revealed posterior displacement of the right colonic flexure and right hepatic lobe, with the liver herniating upward in a 90° angle, extending to the level of the pulmonary hilum (Figure 1).

**Figure 1:**
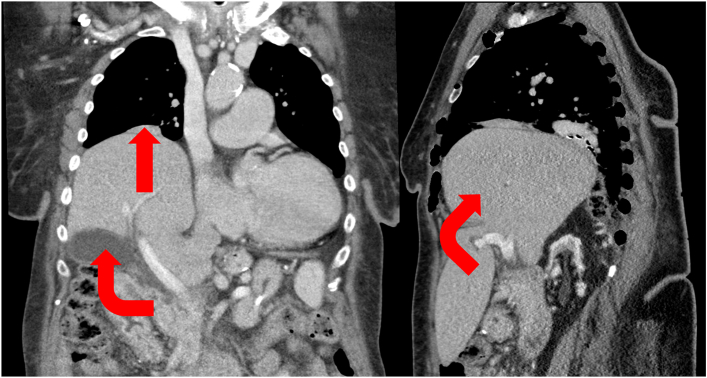
Preoperative portal-venous phase computed tomography (coronal and sagittal reformations) demonstrated the congenital diaphragmatic hernia with herniation of the liver into the thoracic cavity up to the level of the pulmonary hilum (straight arrow). The liver was rotated by approximately 90° (curved arrows), which raised concern for potential compromise of the hepatic hilum during intraoperative reduction.

The patient had significant cardiac comorbidities, including an initial pulmonary artery pressure of 72 mm Hg, atrial fibrillation, and New York Heart Association class I heart failure. She was also diagnosed with class II obesity (body mass index, 39.9 kg/m^2^). Preoperative management was complicated by an upper gastrointestinal bleeding episode, which was successfully treated endoscopically.

Following stabilization, interdisciplinary planning of the operation was carried out with the anesthesiology and radiology services. Given the intrathoracic displacement of the liver, there was concern about potential vascular compromise on hepatic repositioning. Therefore, intraoperative contrast-enhanced ultrasound was scheduled to evaluate hepatic perfusion after reduction.

The patient was positioned in the left lateral decubitus position with the right arm placed anteriorly. Local infiltration was carried out at the 8th intercostal space (ICS) along the posterior axillary line using 3 mL of ropivacaine 0.075%. This was followed by thoracocentesis, placement of the first da Vinci trocar, and introduction of the 30° endoscope. Three additional incisions were made (each with the same local anesthetic infiltration): 7th ICS, anterior axillary line (da Vinci instrument); 9th ICS, scapular line (da Vinci instrument); 10th ICS, midaxillary line (assistant port). The da Vinci Xi robotic system (Intuitive Surgical) was docked without difficulty. Under direct visualization, the robotic instruments were introduced: arm 1, large needle driver; arm 2, 30° endoscope; arm 3, SutureCut; arm 4, offline.

Following insufflation of carbon dioxide, the liver was repositioned spontaneously. The intraoperative findings were consistent with a Bochdalek hernia. To exclude hepatic torsion, sonographic evaluation of liver perfusion was performed (Figure 2).

**Figure 2:**
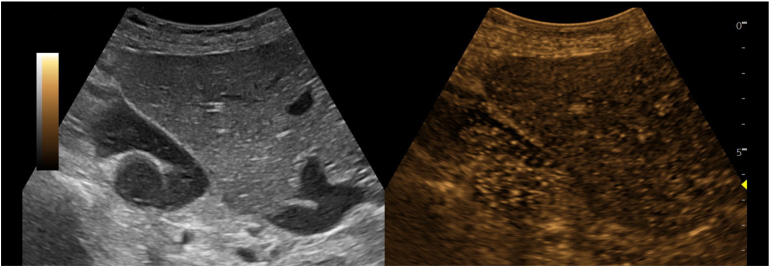
Intraoperative contrast-enhanced ultrasound. Following the intravenous administration of a 1.6 mL bolus of ultrasound contrast agent (SonoVue, Bracco Imaging S.p.A.), the liver demonstrated homogeneous parenchymal enhancement across the entire organ with no perfusion deficits. Furthermore, no focal lesions, regions of portal-venous washout, or signs of thrombosis were evident. Split-screen images are presented with contrast-enhanced ultrasound 80 seconds after injection of the contrast agent on the right and simultaneous corresponding B-mode gray-scale imaging on the left.

The diaphragm was plicated from lateral to medial by a series of 6 U-sutures with Ethibond (Ethicon), reinforced at the U-turns and knot junctions with pledgets. The central suture was placed with FiberWire (Arthrex) to withstand hepatic pressure. For additional reinforcement, a Covidien Parietex mesh (Medtronic) was applied to the plicated diaphragm and secured with individual button sutures. A 20 Ch chest tube was inserted.

Closure of the thoracocentesis sites was performed with running sutures for muscular, subcutaneous, and cutaneous tissue. The chest tube was connected to a suction device (−8 cm H_2_O).

The total operative time was 4 hours and 7 minutes (effective console time, 2 hours). Docking and intraoperative sonography accounted for the remaining 2 hours and 7 minutes. The Video demonstrates the essential operating procedures.

The patient was monitored postoperatively in the intensive care unit. Extubation was successfully performed on postoperative day (POD) 2. The postoperative radiograph is shown in Figure 3. After 5 days in the intensive care unit (respiratory training and regular assessments of hepatic perfusion), the patient was transferred to the general ward. The patient’s subjective dyspnea had significantly improved at this stage. Oxygen supplementation was no longer required, with a stable peripheral oxygen saturation of >95%. The patient was discharged on POD 16 with the chest drain still in place. After outpatient follow-up visits, the drain was removed on POD 30.

**Figure 3:**
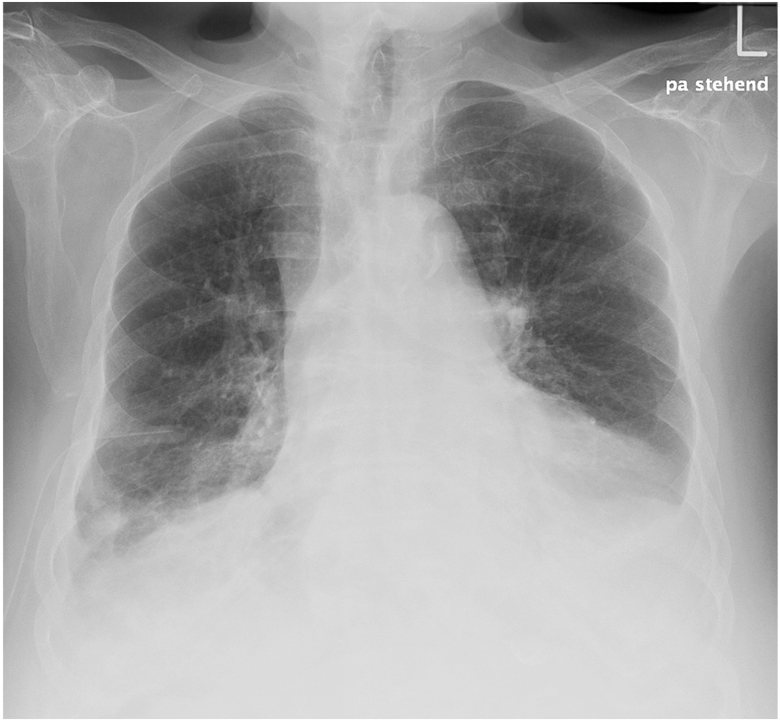
Postoperative radiograph after robot-assisted thoracic surgery diaphragmatic repair. ‘pa stehend’ refers to a posterior–anterior chest X-ray taken in the standing position.

## Comment

This case demonstrates successful robot-assisted thoracic surgery repair of a right-sided Bochdalek hernia with mesh reinforcement in a high-risk patient, highlighting both technical considerations and outcome-specific challenges. The patient’s advanced age, class II obesity, and severe pulmonary hypertension represent significant risk factors for postoperative complications, consistent with literature reporting prolonged recovery in patients with cardiopulmonary comorbidities.^4^^,^^5^

The robotic approach provided enhanced visualization and precision for addressing the intrathoracic liver herniation, corroborating reports that minimally invasive techniques improve outcomes in complex diaphragmatic repairs.^6^ Contrast-enhanced ultrasound to assess hepatic perfusion after reduction represents a prudent adaptation to mitigate risks of vascular compromise, such as liver herniation.^7^ The combination of U-sutures with Ethibond and Parietex mesh reflects a hybrid repair strategy, balancing the durability of primary closure with the reinforcement benefits of synthetic mesh—an approach supported by studies reporting lower recurrence rates with mesh augmentation.^5^^,^^8^

The prolonged pleural effusion requiring chest tube drainage until POD 30 is likely to have stemmed from the patient’s preexisting pulmonary hypertension and extensive diaphragmatic manipulation, a phenomenon observed in cases with significant intrathoracic organ displacement.^4^^,^^5^ The 4-hour operative time exceeds typical laparoscopic durations but remains within reported ranges for robotic repairs of large diaphragmatic defects. Notably, the interdisciplinary preoperative planning underscores the importance of tailored strategies in high-risk cohorts, as emphasized in recent case series.^4^^,^^6^

This case reinforces that robot-assisted thoracic surgery repair with mesh is feasible even in patients with severe cardiopulmonary comorbidities, provided there is intraoperative monitoring and postoperative management.
